# Observations upon the Construction and Use of the Respirator, &c.

**Published:** 1837-04

**Authors:** 


Art. XVII.—*Observations upon the Construction and Use of the Respirator; an Instrument for facilitating the Respiration of Persons suffering under Pulmonary and Bronchial Affections, by Warming the Air inspired, and thereby enabling them to breathe freely in the coldest Atmosphere.* By Julius Jeffreys, Surgeon.—*London,* 1836. 8vo. pp. 12.

No one who has experienced, in his own person, while labouring under catarrhal affections, the effects of sudden exposure to a cold atmosphere, or who has witnessed the common and almost instinctive attempts made by persons so circumstanced to mitigate the painful impression of the air, by means of the handkerchief, &c., will be disposed to dispute the desirableness of any invention calculated to supply a more perfect and effectual defence against a cold atmosphere, in certain circumstances. To one class of invalids in particular, unfortunately not a small one, those persons, namely, who suffer from such extreme sensibility of the glottis and larynx, that they must remain prisoners in the house (although not otherwise sick,) whenever the air sinks below a certain temperature, or there is a considerable degree of wind, an invention of this sort must be invaluable. Whether the instrument described in the pamphlet now before us is calculated to supply the desideratum in question, we have not had sufficient experience to determine; but the result of an examination and temporary trial of the Respirator, leads us to believe that it will be found very useful in many cases, particularly in those last mentioned. We cannot flatter the author with the hope that his instrument will be worn by healthy persons as a preventive of pulmonary disease, nor yet that it will obviate the necessity, as he seems to anticipate, of sending our patients to warmer climates; still, we do believe that it is calculated to be serviceable within certain limits, and therefore recommend it to the attention of the profession.

The principle on which the instrument is constructed is to make the wearer breathe through a congeries of metallic wires, which being warmed by the repeated expirations, give out a constant supply of heat to the air as it is inspired. The following extract from Mr. Jeffreys's pamphlet, explains the particular construction and mode of action of the Respirator; we may add that being small and extremely neat, its appearance is not calculated to deter such as are really invalids from publicly using it; coloured spectacles are only less formidable because familiarised to us by long habit.

"Since the temperature of any one substance must be lower than that of any other before it can receive heat from the latter by conduction, it is plain that a single layer of metal could only take a part of the heat from the breath; even if the contact were of longer duration than it is, the breath would lose no heat after it had raised the metal up to its own temperature. In order to extract more heat from the breath, it must be carried through another layer of metal, which being much colder than itself, can abstract heat from it. As the breath and this second layer approach towards the same temperature, this second layer will not be able to abstract more heat from the breath. It must, therefore, pass through a third still cooler layer, and, for the same reason, through several. In practice, six or eight will not prove too many, and in the coldest weather double that number may be employed. In this series of laminæ, each is warmer than the one in front of it, from the exterior one, which is nearly of the temperature of the atmosphere, to the innermost one, which is perhaps within ten or fifteen degrees of the temperature of the breath. These laminæ would not remain one instant of time at so different states as to heat, if they were placed in contact with each other. In order to preserve the progressive difference in their stocks of heat, they must be kept apart; and it will be found, that during the short period of one respiration, a very small separation will suffice for the purpose. An interval for each of one fortieth of an inch, including the metal, is more than enough; six or eight layers may therefore lie in one-eighth of an inch. These layers having been warmed during an act of expiration, and being each warmer and warmer as they lie nearer the mouth, are enabled to give heat in the most advantageous manner to the fresh air entering from without, which takes up a parcel of heat in traversing each; since, although it grows more and more warm, it is sure to find every layer it comes to warmer than itself, which is, of course, a relative condition necessary for the communication of heat to the air. Not only must the conducting metal be divided into distinct laminæ, that air of different grades of temperature may continue subject to its operation, but in each lamina the metal must be reduced to a state of great extenuation, and be placed in such a manner as to effect the utmost possible division of the breath, so that every particle of it almost may impinge against metal, yet that the passage of the air shall be abundantly free, in order that there may be no perceptible obstruction to the progress of the breath. The Respirator has been constructed with minute attention to the above rules." (P. 7-9.)

